# Self-Photopolymerizable Hydrogel–Ceramic Composites with Scavenger Properties

**DOI:** 10.3390/polym14061261

**Published:** 2022-03-21

**Authors:** Maria Canillas, Gabriel Goetten de Lima, Marcelo J. C. de Sá, Michael J. D. Nugent, Miguel A. Rodríguez, Declan M. Devine

**Affiliations:** 1 Consejo Superior de Investigaciones Cientificas, Instituto de Cerámica y Vidrio, Calle Kelsen, 5, 28049 Madrid, Spain; mar@icv.csic.es; 2 Programa de Pós-Graduação em Engenharia e Ciência dos Materiais—PIPE, Universidade Federal do Paraná, Av. Cel. Francisco H. dos Santos, 100, Jardim das Américas, Curitiba 81530-000, Brazil; ggoetten@research.ait.ie; 3 Materials Research Institute, Technological University of the Shannon, Midlands Midwest, Athlone Campus, University Road, N37 HD68 Athlone, Ireland; mjcdesa@gmail.com (M.J.C.d.S.); michael.nugent@tus.ie (M.J.D.N.); 4 Programa de Pós-Graduação em Medicina Veterinária—PPGMV, Universidade Federal de Campina Grande, Avenida Universitária, s/n, Patos, Santa Cecilia, Sao Paulo 58708-110, Brazil

**Keywords:** photoinitiator, hydrogels, photo-Kolbe reaction

## Abstract

The photocatalytic behaviours of semiconductive ceramic nanoparticles such as TiO_2_, ZnO, Fe_2_O_3_, and Fe_3_O_4_, have been extensively studied in photocatalysis and photopolymerization, due to their ability to produce radical species under ultraviolet–visible light, and even in dark conditions. In addition, in the form of microparticles, TiO_2_ and its Magnéli phases are capable of neutralizing radical species, and a heterogeneous catalytic process has been suggested to explain this property, as it is well known as scavenging activity. Thus, in this study, we demonstrate that these ceramic powders, in the form of microparticles, could be used as photoinitiators in UV polymerization in order to synthesize a hydrogel matrix. Them, embedded ceramic powders could be able to neutralize radical species of physiological media once implanted. The hydrogel matrix would regulate the exchange of free radicals in any media, while the ceramic particles would neutralize the reactive species. Therefore, in this work, the scavenger activities of TiO_2_, ZnO, Fe_2_O_3_, and Fe_3_O_4_ microparticles, along with their photoinitiation yield, were evaluated. After photopolymerization, the gel fraction and swelling behaviour were evaluated for each hydrogel produced with different ceramic initiators. Gel fractions were higher than 60%, exhibiting variation in their scavenging activity. Therefore, we demonstrate that ceramic photoinitiators of TiO_2_, ZnO, Fe_2_O_3_, and Fe_3_O_4_ can be used to fabricate implantable devices with scavenger properties in order to neutralize radical species involved in inflammatory processes and degenerative diseases.

## 1. Introduction

Semiconductive ceramic materials, such as TiO_2_, have been studied in terms of their scavenging activity in order to neutralize radical species in dark conditions [1,2,3], and have also been studied as photocatalysts [4]. Although the exact mechanism of scavenging in this ceramic has not yet been elucidated, Canillas et al. [5] suggested in a previous work that it is derived from a heterogeneous catalytic process, where unsaturated titanium or penta-coordinated titanium in the materials’ surface act as active sites. The geometric configuration of preferential facets in the corresponding ceramic phases plays a key role in the activity; this is due to the accessibility of reactive and solvent molecules to the active site positions. These authors have also shown that ceramic phases of Ti_n_O_n-1_ increase the catalytic activity [6], due to the coexistence of Ti^3+^/Ti^4+^ and the chemistry of defects in their surfaces, allowing charges to be easily transferred through their surfaces. Nevertheless, it is important to mention that the scavenging process requires specific particle sizes; while TiO_2_ microparticles neutralize radical species in dark conditions, nanoparticles are able to produce radical species even in dark conditions, but this provokes genotoxicity and DNA damage by oxidative stress [7,8]. These are the reasons why microparticle size is suggested in the current study, despite nanoparticles being more convenient during the photoinitiation process. The latter is one of the main challenges and contributions of the study presented in this manuscript.

In addition, there are studies of other semiconductive metallic oxides such as ZnO, Fe_2_O_3_, and Fe_3_O_4_ in regards to their photocatalysis and photoinitiation [9,10], as well as their respective scavenging properties [11,12]. Moreover, Fe_3_O_4_ is particularly well known for its photocatalytic properties [13]. Furthermore, Fe_3_O_4_ presents a curious case, and has been studied due to its biological function in the magnetosomes, which consist of Fe_3_O_4_ crystals that are synthetized by magnetic bacteria in order to neutralize intracellular reactive oxygen species effectively [14]. Nevertheless, the photocatalytic properties of these materials have been used to initiate photopolymerization reactions.

The principle behind photopolymerization by these metallic oxides is that these semiconductors, due to their bandgap ability, can induce reactive holes and electrons via UV irradiation, leading to polymerization via photo-Kolbe reaction on acrylates [15]. However, ceramic powders are used at nanoparticle size because of the benefit of these dimensions (surface area x volume), leading to a faster polymerization. The size dependency of the reactivity of ceramic nanoparticles is important [16], as it exhibits a decrease in reactivity, while the particle size increases. In addition, nanoparticles of these ceramics, such as ZnO, prefer to link in acrylate-low rich regions [17]. However, the performance of photocuring from metallic oxides is not sufficient to use in industrial applications [15], and nanoparticles can also have problems of migration. Migration, in this case, refers to any photoinitiator particles that could be transferred through the ambient or the body—the mobility of remaining initiators within the cured polymer—as a form of contamination, and one way to reduce the migration is by increasing the size/mass of the initiator.

These oxides, as initiators in photopolymerization synthesis, can also act as scavengers of radical and reactive species, including DPPH free radicals [12,18,19]. Therefore, they could be used to prepare hydrogel–ceramic composites via photopolymerization, which would be able to neutralize radical species once they are implanted. This is the first time that semiconductive materials have been used with both roles for the same application. In addition, these composites would present several advantages for biomedical applications; the hydrogel matrix would control the exchange of radical species with the surrounding media thanks to its swelling behaviour, with the aim of controlling the levels of radical species within acceptable limits, avoiding harmful rates. Nevertheless, as noted previously, the current process of photopolymerization with these oxide particles involves the usage of nanoparticles, which brings many issues in the fields of biomedical materials and photopolymerization, with the achievement of high yields using microparticles constituting a challenge that researchers have not yet overcome.

In this study, microsized particles of TiO_2_, ZnO, Fe_2_O_3_, and Fe_3_O_4_ ceramic powders were used to synthesize hydrogel–ceramic composites, with the objective to provide an alternative to the already-described harmful nanoparticles and photoinitiator migration problems. These composites can be used to fabricate medical devices, which are able to control reactive oxygen and nitrogen species levels. Control of such species can play a key role in the control of the evolution of diseases such as rheumatoid arthritis (RA), which is an autoimmune disease of unknown aetiology, characterized by chronic inflammation of joints and surrounding tissues, leading to their progressive destruction [20,21,22]. Some studies have evidenced the presence of an increase in the free radical levels of reactive species in RA patients, playing an important role in the aggravation of this disease, in addition to destructive consequences in the inflammation process [23]. Current treatments are fundamentally palliative, and their goals are to prevent joint deterioration, provide pain relief, and maintain the strength and functionality of the joints [24]. The implants developed here could represent a great advance in the treatment of RA.

## 2. Materials and Methods

### 2.1. Materials

The commercial ceramic powders used for this study were ZnO from Sigma-Aldrich, TiO_2_ from Merck, Fe_2_O_3_ from Rectapur, Fe_3_O_4_ from Merck, and ZnO nanoparticles from Merck, all of them with 99% purity.

### 2.2. Polymeric–Ceramic Composition Formulation and Fabrication of Composites

The initial macromonomer solutions and formulations for post-photopolymerization used in this work were polyethylene glycol dimethyl methacrylate (PEGDMA) solution consisting of 67.5 wt% PEGDMA (m.w. 750) and 10 wt% acrylic acid (AA), both from Sigma-Aldrich, 22.5 wt% distilled H_2_O and 0.1 wt% photoinitiator. PEGDMA and AA were dissolved in the corresponding amount of distilled water. After complete dissolution, the photoinitiator was dispersed in the solution by magnetic stirring until complete solubilization. These photoinitiators were from ceramic powders of ZnO, TiO_2_, Fe_2_O_3_, and Fe_3_O_4_ in micrometric grain sizes. Nanometric ZnO was used to compare the photoinitiation activity effect of a micro- to a nano-formulation. Moreover, 4-(2-hydroxyethoxy)phenyl-(2-hydroxy-2-propyl)ketone, Irgacure 2959 (from Ciba Specialty Chemicals), was used as a photoinitiator control [25]. The PEGDMA-based solution was divided into aliquots, which were placed in different cavities of a silicon mould. Subsequently, UV curing was achieved following 45 min in a UV curing system (Dr. Gröbel UV-Electronik GmbH). The irradiation chamber utilized was a controlled radiation source with 20 UV tubes that provide a spectral range between 315 and 400 nm at an average intensity of 10–13.5 mW/cm^2^.

### 2.3. Physicochemical Characterization of Ceramic Powders and Composites

X-ray diffraction (XRD) analyses were performed on the ceramic powders using a Bruker D8 Advance diffractometer with Cu Kα radiation and a LynxEye^®^ detector, with a θ–2θ configuration in the range of 10 to 70°. In case of samples containing Fe, the detector discrimination settings must be adjusted in order to avoid the range in which the fluorescence signal is collected. The width of the window is narrowed to between 0.18 and 0.036. The particle size distributions of these powders were measured by laser scattering, using a Mastersizer S (Malvern Panalytical Ltd., Malvern, UK). The specific surface areas were measured using a Monosorb MS 13. (Quantachrome, Boynton Beach, FL, USA). The bandgaps of the semiconductive ceramic powders were determined from the Kubelka–Munk transformation of corresponding ultraviolet–visible (UV–Vis) spectra. Absorbance spectra were obtained by using a Lambda 950 UV–Vis–NIR spectrophotometer (PerkinElmer, Waltham, MA, USA). The measurements were carried out in the diffuse reflectance (DR) mode. The bandgap was calculated using the equation according to van Leeuwen et al., where the absorption coefficient (α) is related to the photon energy (hν) [26].

To characterize the polymeric–ceramic composites, attenuated total reflectance Fourier-transform infrared spectroscopy (ATR-FTIR) was used with a PerkinElmer (Spectrum One, Waltham, MA, USA) spectrometer fitted with a universal ATR sampling accessory. All data were recorded in the spectral range of 400–4000 cm^−1^.

### 2.4. Scavenging Activity Assays

The 1,1-diphenyl-2-picryl-hydrazyl radical (DPPH·) in 2-propanol was used to study the scavenging activity of the proposed samples. DPPH· is a semi-stable radical, which absorbs at λ = 517 nm (purple colour), and in the presence of anti-oxidative agents in 2-propanol the radical is neutralized as DPPH_2_. The absorption vanishes as the electrons pair off; the resulting decolouration is stoichiometric with respect to the number of electrons occupied. The DPPH· concentration can be easily measured by UV–Vis absorbance, aided by a calibration curve. The difference between the initial and the remaining concentrations of DPPH·, after the incubation time, will give the percentage of DPPH· eliminated [27].

The materials studied had different specific surface areas, which affect the kinetics of the reaction. For this reason, the same ratio of specific surface area to volume of DPPH· solution was used in all conditions, corresponding to 1 m^2^ surface area of different ceramic powders per 1 mL of 0.1 mM DPPH· solution. Incubation was carried out in dark conditions in order to avoid scavenging by photocatalysis, for 3 and 24 h. Then, the suspension was centrifuged, and the absorbance of the supernatant solutions was measured with a UV-Vis-NIR spectrophotometer (PerkinElmer, Lambda 950). Subsequently, the percentage of DPPH· eliminated was calculated from the ratio between the initial and final concentrations.

### 2.5. Swelling and Gel Fraction

After photopolymerization, samples were immersed in a buffer solution (pH 7.4) to determine their water uptake. Samples were removed after 24 h and their weight was measured. The equilibrium water content (EWC) was calculated using Equation (1):

(1) $$\mathrm{EWC}\;\left(\%\right)=\frac{W_{s}-{\;W}_{d}}{W_{d}}*100\;$$

where W_d_ and W_s_ are the weights of the composites after photopolymerization when dried and in the swollen state, respectively. Results are expressed as the average of 5 samples (±SD) [28].

The gel fraction was calculated from the weight of dried samples before and after swelling, according to Equation (2):

(2) $$\%G\;=\frac{W_{d}}{W_{\mathrm{ds}}}\;*100$$

where W_d_ represents the weight of the dried composites before swelling, and W_ds_ represents the composite weight after swelling for 24 h [28].

Furthermore, in this study, the ability of the different ceramic powders to promote jellification was studied and compared between samples. To this end, the percentages of gel fractions were divided by the corresponding surface area to determine the jellification capability of different ceramic materials per m^2^ of powder surface.

## 3. Results and Discussion

### 3.1. Physicochemical Characterization of Ceramic Powders

**Figure 1:**
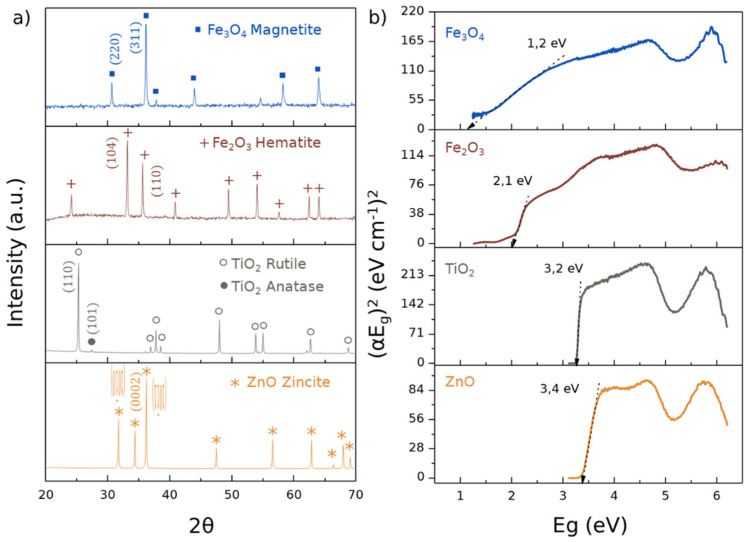
Characterization of semiconductive ceramic powders: TiO_2_, ZnO, Fe_2_O_3_, and Fe_3_O_4_. (**a**) The X-ray diffractions showing the phase compositions; (**b**) Kubelka–Munt calculations from the UVA–Vis spectra, also exhibiting bandgap values (eV).

The phase compositions of the commercial ceramic powders used for this study were characterized by XRD (Figure 1a), and the ceramics ZnO, Fe_2_O_3_, and Fe_3_O_4_ were each composed of a single phase, corresponding to zincite, hematite, and magnetite, respectively. However, in the case of TiO_2_, it corresponded to a mixture of a majority phase of rutile and a minority phase of anatase.

These metal ceramic oxides were characterized by their bandgap energies, owing their photocatalytic activity to their semiconductive nature, whereas photoexcited electrons were promoted from the valence band to the conduction band. This interaction forms an electron–hole pair (e^−^/h^+^), which is able to reduce and/or oxidize a compound while also being able to adsorb on the photocatalytic surface. The bandgaps of these oxides were obtained from the Kubelka–Munt calculations of the corresponding UV–Vis spectra, as shown in Figure 1b. ZnO and TiO_2_ exhibited similar values, corresponding to 3.4 and 3.2 eV, respectively; however, these values were reduced in the case of Fe_2_O_3_ and Fe_3_O_4_, to 2.1 and 1.2 eV, respectively.

Other important parameters in the photocatalytic, but also catalytic, processes include the particle size and specific surface area (SSA). For this reason, both were measured (Table 1), and the values were correlated with the photoinitiation processes and their scavenging activities.

As expected, the specific surface area increased when the particle size decreased. In the case of ZnO, the SSA drastically decreased compared to Fe_3_O_4_, with similar particle sizes; however, the SSA was quite similar to that of Fe_2_O_3_, but with a considerably larger particle size.

### 3.2. Scavenging Activity

**Figure 2:**
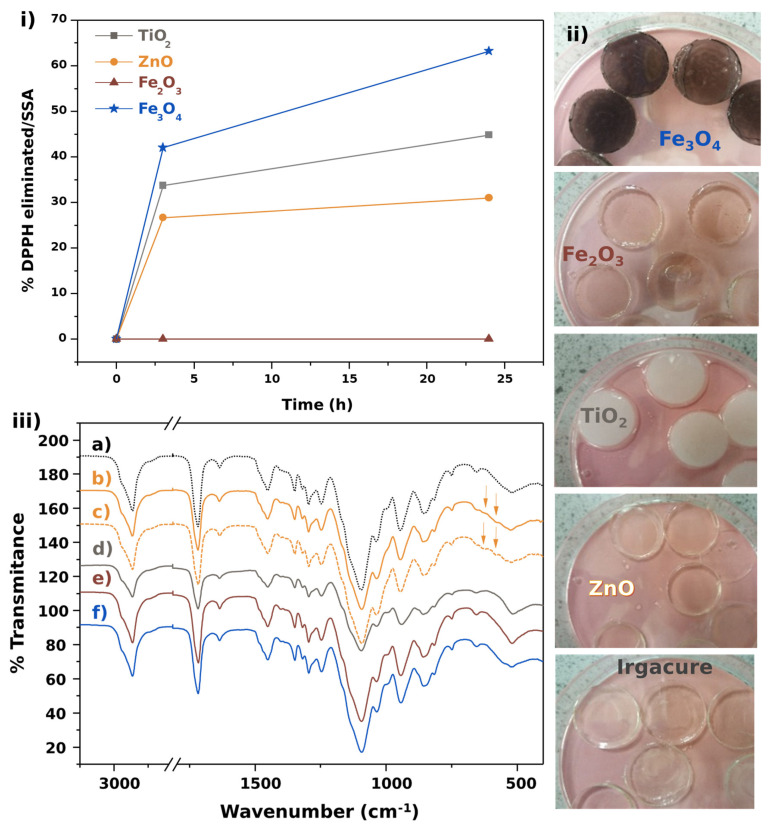
(**i**) Scavenging activity of various ceramic powder materials after 3 and 24 h; (**ii**) PEGDMA-based compositions using Irgacure and ZnO, TiO_2_, Fe_2_O_3−_, and Fe_3_O_4_ as photoinitiators; (**iii**) FTIR of PEGDMA-based composites synthesized by photopolymerization using different photoinitiators: (a) Irgacure, (b) ZnO, (c) nano-ZnO, (d) TiO_2_, (e) Fe_2_O_3_, and (f) Fe_3_O_4_.

The scavenging activity of the ceramic oxides was evaluated by the amount of DPPH· consumed after 3 and 24 h (Figure 2i) and, in order to compare the scavenging activity of different materials, the scavenging activity is shown as % of DPPH eliminated per m^2^ of SSA, as in previous studies [5,6]. Ceramic oxide of Fe_3_O_4_ exhibited the highest scavenging activity, while Fe_2_O_3_ presented a lack of scavenging capability. Between both scavenging activities, from ZnO and TiO_2_, the values of scavenging activities increased in the following order: ZnO < TiO_2_ < Fe_3_O_4_.

According to previous studies, the ability of TiO_2_ to neutralize radical species follows a catalytic mechanism, where the unsaturated metal sites of the surfaces act as active centres in which solvent or radical molecules are adsorbed. The geometry of the preferential facets in the ceramic surfaces, along with the position of active centres in these facets, determines the higher or lower disposition of active points to interact with the solvent or dissolved molecules, as well as the stability of the links between ceramic surfaces and molecules in solution. For example, TiO_2_ presents two stable phases—rutile and anatase—which exhibit different preferential facets and, consequently, different catalytic activities. Both phases have facets with unsaturated Ti, or penta-coordinated Ti (Ti_5c_), in their surfaces, which act as reactive sites. The geometry of the facets and, consequently, the availability of Ti_5c_ sites, will determine the effectiveness of the catalytic process. Therefore, modelling of water molecules’ adsorption from different surface structures of TiO_2_ indicates that rutile (110) is more stable than (101) anatase surfaces. The position of Ti_5c_ in rutile seems to favour the adsorption of solvent molecules compared with anatase. Since commercial powders used in this study show rutile as the majority phase [29,30,31], this would explain its high catalytic activity.

The reactivity of zincite also depends on the geometric configuration of the preference facets [32], as well as the space occupied by the corresponding active sites, unsaturated tetragonal Zn, or Zn_3c_ in their surfaces. The crystal structure of this ceramic oxide is the hexagonal system, and it shows $\left\{10\bar{1}0\right\}$ planes in parallel to the c-axis [0001] direction, and is limited from the top and the bottom by {0001} planes. The relative intensity of $\left(10\bar{1}0\right)$ to (0002) indicates that there is a preferential crystallographic orientation. The zincite diffractogram exhibited a higher ratio intensity from I($10\bar{1}0$)/I(0002), which was also related to a higher number of {0001} planes. Therefore, {0001} planes presented a suitable scavenger activity, albeit lower than that of rutile and its (110) facets.

Finally, the studied iron oxides presented different activities. While Fe_2_O_3_ does not present scavenging activity, Fe_3_O_4_ had the highest scavenging activity among the studied oxides. In the case of hematite, or α-Fe_2_O_3_, it crystallizes in the rhombohedral system, and Fe(III) atoms are located in the tetrahedral sites. Unsaturated Fe_3c_ contains the reactive sites where molecules adsorb, catalysing the corresponding reaction. The ratio corresponding to I_(104)_/I_(110)_ is related to the exposed {012} and {001} facets. Studies have shown that nanoparticles of Fe_3_O_4_ with preferentially exposed {012} facets exhibit higher catalytic activities compared with {001} facets. Therefore, commercial Fe_2_O_3_ powders had a higher number of exposed {001} [33], and this parameter could be the reason that explains the absence of scavenging activity in this sample, in addition to its high particle size.

In the case of magnetite, it presented the best scavenging activity results, crystallizing as a spinel in an fcc system. The Fe atoms were arranged in tetrahedral sites, with oxidation state (III), as well as octahedral sites with oxidation states (II) and (III). Studies have shown that the adsorption of methanol molecules in the step edges of the (001) surface of magnetite, along the $\left[0\bar{1}1\right]$, exposes Fe_tet_ atoms with only one dangling bond; however, along the [110], exposures occurred of Fe_oct_ and Fe_tet_, with three and two dangling bonds, respectively [34]. The higher the undersaturation, the higher the reactivity, so the undersaturation in these step edges would explain the higher scavenging activity observed.

Another important parameter is the specific surface area. In all cases, higher specific surface area contributed to a higher reactive area and, consequently, higher scavenging activities. However, if we compare the SSA provided in Table 1 and the scavenging activity curves shown in Figure 2i, it seems that scavenging activity is not directly related to SSA. Therefore, the crystal structure systems in the surfaces must play a more dominant and crucial role.

Since all of these ceramic oxides present scavenging activity in dark conditions, but photocatalytic properties under light conditions, they can be used as photoinitiators in order to obtain polymeric–ceramic composites with many applications, such as in medical devices for the control of ROS and RNS.

The composites prepared can be observed in Figure 2ii, exhibiting the various colours transmitted from the samples obtained using the various initiators. An interesting case among these materials is that of ZnO, since it is transparent, but its powder has a white appearance; however, it exhibited some scavenging activity, so if a material is required to preserve the transparency of the composite, ZnO can be a good alternative.

FTIR spectra (Figure 2iii) exhibit the polymerization reaction using different ceramic photoinitiators for the PEGDMA network. The main bands of this hydrogel were assigned (Appendix A Table A1) to its typical vibration modes [35]. The composite fabricated with ZnO nanoparticles was unique, as it was the only one that exhibited a shift from the band at 1093 cm^−1^ to 1097 cm^−1^. This could be attributed to a stronger interaction between the OR groups and the ZnO nanoparticle surfaces. Moreover, the spectra show shoulders at 1167 and 615 cm^−1^, and these signals are due to the unpolymerized PEG macromonomers.

**Figure 3:**
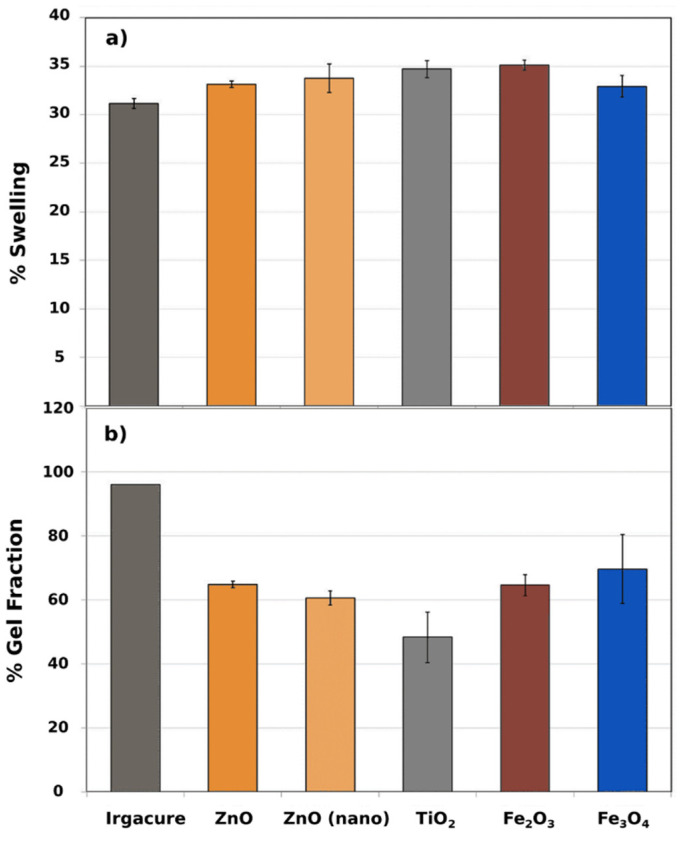
(**a**) Swelling percentages of the composites photoinitiated using Irgacure and different ceramic powders (ZnO, nano-ZnO, TiO_2_, Fe_2_O_3_, and Fe_3_O_4_); (**b**) gel fraction percentages of the composites photoinitiated using Irgacure and different ceramic powders (ZnO, nano-ZnO, TiO_2_, Fe_2_O_3_, and Fe_3_O_4_).

The swelling percentage and the gel fraction were measured in order to study the gel networks obtained with different photoinitiators, as well as their exchange capacity within the media. The different composites exhibited swelling percentages between 30 and 35%. (Figure 3a) No significant differences were perceived between the composites fabricated with the studied ceramics as photoinitiators, implying that the exchange capacity to recruit medium to the PEGDMA matrix is similar in all cases.

Considering the gel fraction percentages, materials photoinitiated by ceramic powders present lower values compared to Irgacure (Figure 3b). Although Irgacure is an effective photoinitiator, microparticles of ceramic powders are also capable of photoinitiating the polymerization process. However, no significant differences were observed between the studied composites photoinitiated with various ceramics. The composite prepared from TiO_2_ exhibited a slightly lower gel fraction. In addition, we observed similar gel fractions obtained for composites obtained from micrometric and nanometric ZnO powders, exhibiting the same efficiency, and using the same wt%, but different specific surface areas.

To compare the abilities of the different ceramics to photoinitiate the polymerization reaction, percentages of gel fraction were studied per unit of specific surface area (SSA) and compared between them; therefore, each composite with a ceramic photoinitiator gel fraction was divided by its SSA. The principle behind photoinitiation is related to a catalytic process that takes place in the surface of the ceramic powders. Since different ceramic powders were added at the same weight, and they possess different surface areas, this parameter must be considered in order to effectively carry out the comparative study.

**Figure 4:**
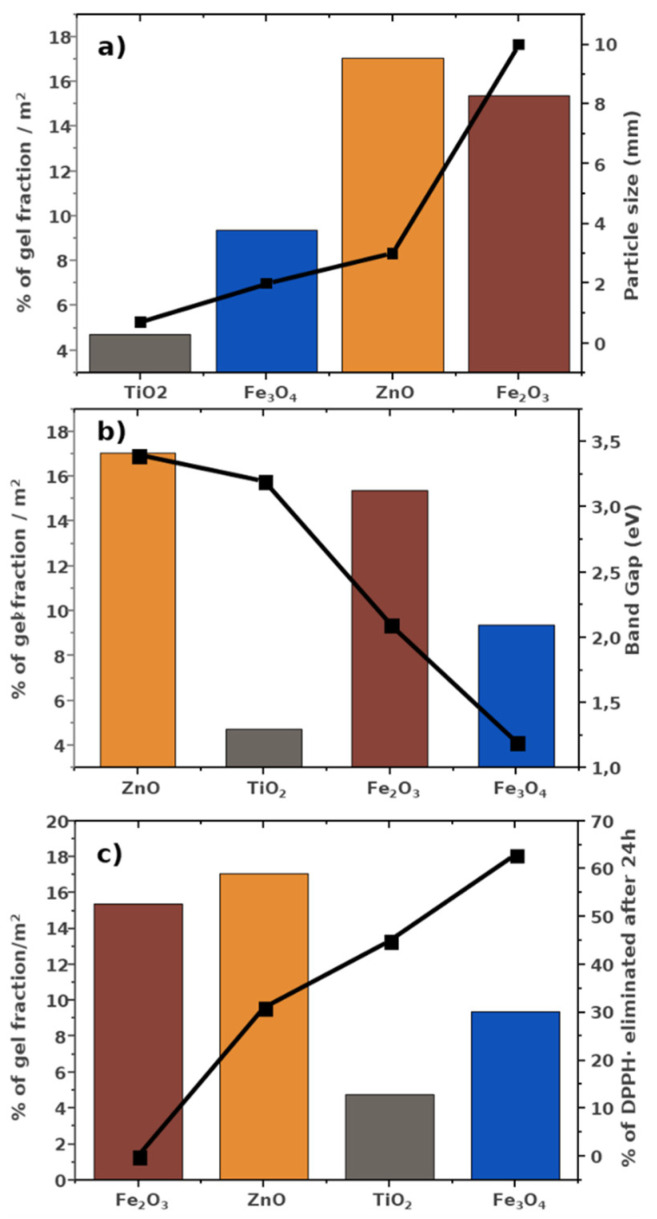
Gel fraction percentages of the composites photoinitiated with ceramic powders (ZnO, nano-ZnO, TiO_2_, Fe_2_O_3_, and Fe_3_O_4_) per unit of ceramic specific surface area (bars) vs. the tendency of (**a**) particle size, (**b**) bandgap values, and (**c**) scavenging activity (dotted lines).

Since initiation is related to the photocatalytic activity, it is possible to relate the gel fraction percentage to bandgap values, the specific surface area and, consequently, the particle size. However, it does not appear that any of these parameters follows the trend of the gel fraction (Figure 4a,b). It is important to recall that there is a relationship between photocatalysis and the reactive sites, related to the unsaturated metallic atoms (Ti_5c_, Zn_3c_, Fe_5c_, or Fe_3c_). However, in this study, the scavenging activities and the gel fractions per m^2^ of specific surface area did not seem to be correlated (Figure 4c). Therefore, it is possible that another key parameter could be involved in the efficiency of photoinitiation.

When the gel fraction percentages (per m^2^ of specific surface of the photoinitiator) are compared with their visual observations (Figure 3b), it can be concluded that the optical properties of the samples are a key parameter during photopolymerization. The samples that presented the lowest percentage of gel fraction/m^2^ of specific surface area presented opacity, such as TiO_2_ powders, in composites of which light is reflected and dispersed. The sample with the second lowest gel fraction percentage was the Fe_3_O_4_ powder, which showed translucent properties. Finally, the samples with the highest gel fraction percentage were the composites with Fe_2_O_3_ and ZnO, which were completely transparent. TiO_2_ and Fe_3_O_4_ were the powders with the lowest particle size and the highest specific surface area and, when they were dispersed in the monomer solution, they promoted the dispersion and reflection of the light, decreasing the efficiency of the photopolymerization process.

## 4. Conclusions

The semiconductive ceramic microparticulate powders studied in this work—TiO_2_, ZnO, Fe_2_O_3_, and Fe_3_O_4_—were able to photoinitiate the photopolymerization reaction, obtaining a hydrogel matrix with a gel fraction of around 60%. TiO_2_ powders gave the lowest gel fraction percentage—around 50%. Despite Irgacure being more effective in the photoinitiation process, microparticles prevented secondary problems after implantation, and possess some advantages for medical applications, such as their scavenging activity.

The oxides TiO_2_, ZnO, and Fe_3_O_4_ exhibited scavenging properties with different capabilities. From the highest, around 60% of radicals were neutralized for Fe_3_O_4_, and around 40% and 30%, for TiO_2_ and ZnO, respectively. Fe_2_O_3_ did not show scavenging activity. Therefore, Fe_3_O_4_ could be a good candidate for the application suggested here.

The photoinitiation activity of the powders is related to the photoactivity of these semiconductive ceramic powders, while the scavenging activity is related to a heterogeneous catalytic process. In this case, the properties of the surface, which are intimately related to the catalytic processes, are responsible for the scavenging effectiveness. However, in the case of photoinitiation, the optical properties of the composite also seem to play a key role in the success of the jellification reaction.

## Figures and Tables

**Table 1:** Physicochemical properties of the semiconductive ceramic powders. Particle size distribution and specific surface area.

Materials	Particle Size (µm)	Specific Surface Area (m^2^/g)
ZnO	3	3.8
TiO_2_	0.7	10.2
Fe_2_O_3_	10	4.2
Fe_3_O_4_	2	8.7

## Data Availability

Data will be made available upon request.

## Appendix A

**Table A1:** FTIR bands allocation.

Band Assignment	Wavenumber (cm^−1^)
OH stretching	3569
CH stretching	2867
C=C bending	1721 and 1638
CCH_2_ bending	1452
CH vibration of CH_3_ groups	1349
OR stretching	1093

## References

[1] De Pasquale I., Lo Porto C., Dell’Edera M., Petronella F., Agostiano A., Curri M.L., Comparelli R. (2020). Photocatalytic TiO_2_-Based Nanostructured Materials for Microbial Inactivation. Catalysts.

[2] Suzuki R., Muyco J., McKittrick J., Frangos J.A. (2003). Reactive oxygen species inhibited by titanium oxide coatings. J. Biomed. Mater. Res..

[3] Sahlin H., Contreras R., Gaskill D.F., Bjursten L.M., Frangos J.A. (2006). Anti-inflammatory properties of micropatterned titanium coatings. J. Biomed. Mater. Res. Part A.

[4] Rajh T., Dimitrijevic N.M., Bissonnette M., Koritarov T., Konda V. (2014). Titanium Dioxide in the Service of the Biomedical Revolution. Chem. Rev..

[5] Canillas M., Chinarro E., Freitas M., Pêgo A.P., Moreno B. (2020). Titanium dioxide catalytic activity contributes to the process of free radical scavenging. J. Catal..

[6] Canillas M., Chinarro E., Pêgo A.P., Moreno B. (2017). Scavenging activity of Magnéli phases as a function of Ti 4+ /Ti 3+ ratios. Chem. Commun..

[7] Trouiller B., Reliene R., Westbrook A., Solaimani P., Schiestl R.H. (2009). Titanium Dioxide Nanoparticles Induce DNA Damage and Genetic Instability In vivo in Mice. Cancer Res..

[8] Damm C., Völtzke D., Abicht H.-P., Israel G. (2005). Influence of the properties of TiO_2_ particles on a photocatalytic acrylate polymerisation. J. Photochem. Photobiol. A Chem..

[9] Stroyuk A.L., Sobran I.V., Kuchmiy S.Y. (2007). Photoinitiation of acrylamide polymerization by Fe_2_O_3_ nanoparticles. J. Photochem. Photobiol. A Chem..

[10] Ulku I., Morlet-Savary F., Lalevée J., Yagci Acar H. (2018). Homogenous photopolymerization of acrylic monomers initiated with ZnO-methacrylate in non-aqueous medium and production of luminescent nanocomposites. Polym. Chem..

[11] Moskvin M., Horák D. (2016). Carbohydrate-Modified Magnetic Nanoparticles for Radical Scavenging. Physiol. Res..

[12] Marin-Flores C.A., Rodríguez-Nava O., García-Hernández M., Ruiz-Guerrero R., Juárez-López F., Morales-Ramírez A.d.J. (2021). Free-radical scavenging activity properties of ZnO sub-micron particles: Size effect and kinetics. J. Mater. Res. Technol..

[13] Dadashi-Silab S., Yar Y., Yagci Acar H., Yagci Y. (2015). Magnetic iron oxide nanoparticles as long wavelength photoinitiators for free radical polymerization. Polym. Chem..

[14] Guo F.F., Yang W., Jiang W., Geng S., Peng T., Li J.L. (2012). Magnetosomes eliminate intracellular reactive oxygen species in Magnetospirillum gryphiswaldense MSR-1. Environ. Microbiol..

[15] Schmitt M. (2012). ZnO Nanoparticle Induced Photo-Kolbe Reaction, Fragment Stabilization and Effect on Photopolymerization Monitored by Raman-UV-Vis Measurements. Macromol. Chem. Phys..

[16] Ramakrishna G., Ghosh H.N. (2003). Effect of Particle Size on the Reactivity of Quantum Size ZnO Nanoparticles and Charge-Transfer Dynamics with Adsorbed Catechols. Langmuir.

[17] Goourey G.G., de Sainte Claire P., Balan L., Israëli Y. (2013). Acrylate photopolymer doped with ZnO nanoparticles: An interesting candidate for photo-patterning applications. J. Mater. Chem. C.

[18] Brezová V., Dvoranová D., Staško A. (2007). Characterization of titanium dioxide photoactivity following the formation of radicals by EPR spectroscopy. Res. Chem. Intermed..

[19] Yulizar Y., Kusrini E., Apriandanu D.O.B., Nurdini N. (2020). *Datura metel* L. Leaves extract mediated CeO_2_ nanoparticles: Synthesis, characterizations, and degradation activity of DPPH radical. Surf. Interfaces.

[20] Pham-Huy L.A., He H., Pham-Huy C. (2008). Free radicals, antioxidants in disease and health. Int. J. Biomed. Sci..

[21] Rose N.R., Mackay I.R. (2006). The Autoimmune Diseases.

[22] Rawla P. (2019). Cardiac and vascular complications in rheumatoid arthritis. Reumatologia/Rheumatology.

[23] Hadjigogos K. (2003). The role of free radicals in the pathogenesis of rheumatoid arthritis. Panminerva Med..

[24] Laires P.A., Laíns J., Miranda L.C., Cernadas R., Rajagopalan S., Taylor S.D., Silva J.C. (2017). Inadequate pain relief among patients with primary knee osteoarthritis. Rev. Bras. Reumatol..

[25] Killion J.A., Geever L.M., Devine D.M., Farrell H., Higginbotham C.L. (2014). Compressive Strength and Bioactivity Properties of Photopolymerizable Hybrid Composite Hydrogels for Bone Tissue Engineering. Int. J. Polym. Mater. Polym. Biomater..

[26] Van Leeuwen R.A., Hung C.-J., Kammler D.R., Switzer J.A. (1995). Optical and Electronic Transport Properties of Electrodeposited Thallium(III) Oxide Films. J. Phys. Chem..

[27] Suzuki R., Frangos J.A. (2000). Inhibition of Inflammatory Species by Titanium Surfaces. Clin. Orthop. Relat. Res..

[28] de Lima G.G., Campos L., Junqueira A., Devine D.M., Nugent M.J.D. (2015). A novel pH-sensitive ceramic-hydrogel for biomedical applications. Polym. Adv. Technol..

[29] Diebold U. (2003). Structure and properties of TiO_2_ surfaces: A brief review. Appl. Phys. A Mater. Sci. Process..

[30] Pan J., Liu G., Lu G.Q.M., Cheng H.-M. (2011). On the True Photoreactivity Order of {001}, {010}, and {101} Facets of Anatase TiO_2_ Crystals. Angew. Chemie Int. Ed..

[31] Futera Z., English N.J. (2017). Exploring Rutile (110) and Anatase (101) TiO 2 Water Interfaces by Reactive Force-Field Simulations. J. Phys. Chem. C.

[32] Liu X., Ye L., Liu S., Li Y., Ji X. (2016). Photocatalytic Reduction of CO2 by ZnO Micro/nanomaterials with Different Morphologies and Ratios of {0001} Facets. Sci. Rep..

[33] Xiang Q., Chen G., Lau T.-C. (2015). Effects of morphology and exposed facets of α-Fe_2_O_3_ nanocrystals on photocatalytic water oxidation. RSC Adv..

[34] Gamba O., Hulva J., Pavelec J., Bliem R., Schmid M., Diebold U., Parkinson G.S. (2017). The Role of Surface Defects in the Adsorption of Methanol on Fe_3_O_4_(001). Top. Catal..

[35] de Lima G.G., Elter J.K., Chee B.S., Magalhães W.L.E., Devine D.M., Nugent M.J.D., de Sá M.J.C. (2019). A tough and novel dual-response PAA/P(NiPAAM-co-PEGDMA) IPN hydrogels with ceramics by photopolymerization for consolidation of bone fragments following fracture. Biomed. Mater..

